# The Mediating Role of Satisfaction with Life and Social Interaction Anxiety in the Relationship Between Loneliness and Regulatory Emotional Self-Efficacy

**DOI:** 10.3390/bs15030392

**Published:** 2025-03-20

**Authors:** Juncheng Guo, Mansor Abu Talib, Bolin Guo, Jiaxin Ren, Jia Liu

**Affiliations:** 1 Faculty of Social Sciences and Liberal Arts, UCSI University, Kuala Lumpur 56000, Malaysia; jiaxin0776@gmail.com; 2 Wellbeing Research Centre, UCSI University, Kuala Lumpur 56000, Malaysia; mansorat@ucsiuniversity.edu.my; 3 Human Resources Education and Training Centre, Ganzhou Bank, Ganzhou 341000, China; 13763940509@163.com; 4 School of Foreign Languages, Gannan Normal University, Ganzhou 341000, China; 18970131585@163.com

**Keywords:** loneliness, satisfaction with life, social interaction anxiety, regulatory emotional self-efficacy, mental health, well-being

## Abstract

Regulatory emotional self-efficacy represents individuals’ belief in their capacity to manage emotions effectively and plays a vital role in supporting emotional well-being and adaptive functioning, particularly in university students. This study explores the influence of loneliness on regulatory emotional self-efficacy and its underlying mechanisms by incorporating two mediating variables—satisfaction with life and social interaction anxiety—within a chain mediation model. A total of 547 undergraduate students from a university located in Nanchang, Jiangxi Province, participated in the survey. The findings reveal that loneliness directly impacts regulatory emotional self-efficacy; satisfaction with life mediates the effect of loneliness on regulatory emotional self-efficacy; social interaction anxiety also mediates the effect of loneliness on regulatory emotional self-efficacy; and satisfaction with life and social interaction anxiety jointly serve as chain mediators in the relationship between loneliness and regulatory emotional self-efficacy. This study sheds light on the connection between loneliness and regulatory emotional self-efficacy, offering a theoretical basis and practical guidance for improving students’ emotional regulation and overall well-being.

## 1. Introduction

Emotional regulatory self-efficacy (ERSE), referring to the confidence in one’s capacity to effectively manage and regulate emotions, has become an increasingly important factor in higher education (G. V. Caprara et al., 2008). College students face various stressors, including academic challenges, social pressures, and significant life transitions, all of which require strong emotional regulation skills (Karyotaki et al., 2020). Mental health disorders are prevalent among this population, with anxiety, depression, and stress being particularly common (Ramón-Arbués et al., 2020). Epidemiological studies indicate that a significant proportion of college students experience psychological distress, often exacerbated by academic performance concerns, financial instability, and social adaptation difficulties (Beiter et al., 2015). Emerging research has also identified trauma and borderline personality traits as contributing factors to mental health challenges in young adults, further complicating their ability to manage stress effectively (Malafanti et al., 2024). Additionally, sleep disorders are frequently reported among undergraduate students, which can negatively impact cognitive functioning and emotional regulation (Zafar & Ansari, 2020). Other psychosocial factors, such as the increasing burden of digital engagement and the uncertainties surrounding post-graduation career prospects, have been linked to heightened stress levels and depressive symptoms in this demographic (Acharya et al., 2018). Suicide risk is another critical concern, with studies highlighting clinical predictors that may help in early identification and intervention among at-risk college students (Gómez, 2020). Understanding ERSE can provide insights into the development of effective intervention programs that enhance students’ emotional skills, promoting mental health and potentially reducing dropout rates (Bacon et al., 2018). In the following section, we will discuss key concepts and the theoretical framework underlying this study.

## 2. Key Concepts and Theoretical Framework

Current research on the factors affecting emotional self-regulation in individuals often emphasizes two primary domains: personal psychological attributes and external social conditions (Kleef, 2009; Pocnet et al., 2017). Within this framework, increasing attention is directed towards the notable impact of social factors, such as family and peer relationships, demonstrating a positive association between strong social support networks and effective emotional regulation (Cui et al., 2020). Further studies have affirmed that an individual’s social surroundings, particularly the quality of interpersonal connections, serve as critical predictors of emotional self-efficacy (Elegbede & Ogunleye, 2018; Loeb et al., 2016). However, the role of loneliness, a variable directly impacting emotional well-being, has emerged as a significant yet underexplored factor influencing self-regulation efficacy (Dagan & Yager, 2019; Tu & Zhang, 2015).

Loneliness refers to an individual’s subjective sense of social isolation, marked by perceived inadequacies in forming meaningful connections and the absence of sufficient social support (Fromm-Reichmann, 1990). This construct has been widely examined in psychological research, revealing that loneliness encompasses emotional, cognitive, and relational dimensions that impact one’s self-perception and mental well-being (Hyland et al., 2019). Self-Determination Theory (SDT), which highlights the significance of meeting psychological needs—particularly the need for relatedness—for optimal functioning and well-being, also provides support for the detrimental effects of loneliness on regulatory emotional self-efficacy (RESE; Ryan & Deci, 2000). According to SDT, the experience of loneliness signifies an unmet need for social connection, which in turn weakens one’s resilience and capacity to regulate emotions effectively (Vansteenkiste & Ryan, 2013).

Regulatory emotional self-efficacy, on the other hand, denotes an individual’s confidence in their capacity to handle and regulate emotions in various circumstances (Cacioppo et al., 2002). This construct is critical for psychological resilience, as it enables individuals to maintain emotional stability and respond adaptively to stress (Wu et al., 2022). Rooted in Social Cognitive Theory (SCT), the development of emotional self-efficacy relies on positive social interactions and reinforcement, which help build confidence in emotional control (Schunk & DiBenedetto, 2020). However, for individuals experiencing loneliness, these essential social reinforcements are often absent, leading to diminished regulatory emotional self-efficacy (Hayes et al., 2022).

## 3. The Role of Emotions and Cognition in Self-Efficacy

Emotions influence not only self-efficacy but also decision-making, memory, attention, and judgment (Mayiwar et al., 2024). Affect-as-Information Theory (Clore et al., 2012; Van Lange et al., 2012) suggests that individuals use their emotional states as a source of information when making judgments about their abilities. For instance, individuals who frequently experience negative emotions, such as loneliness or sadness, may interpret these emotions as indicators of incompetence, leading to a self-reinforcing negative feedback loop (Meng et al., 2020). Emotional Reasoning Theory (Gangemi et al., 2013) further posits that emotions shape self-perceptions in a recursive manner, reinforcing beliefs about personal efficacy. For example, a cycle may emerge where loneliness leads to lower self-efficacy, which further exacerbates feelings of loneliness, perpetuating emotional distress and psychological dysfunction (Hladek et al., 2019).

Satisfaction with life is another crucial factor influencing regulatory emotional self-efficacy (M. Caprara et al., 2020). It reflects an individual’s cognitive evaluation of their overall quality of life and well-being (Diener et al., 1985). Lower life satisfaction has been linked to emotional instability and a weakened sense of personal agency (Veronese et al., 2019). Moreover, social interaction anxiety—characterized by excessive fear and apprehension in social settings—has been shown to negatively impact emotional self-regulation (Tsarpalis-Fragkoulidis et al., 2022). Individuals experiencing heightened social interaction anxiety may struggle with forming meaningful social connections, thereby reinforcing loneliness and reducing their perceived ability to regulate emotions effectively (Robert Eres et al., 2021).

## 4. The Role of Social Interaction Anxiety in Self-Efficacy

Social interaction anxiety is characterized by excessive fear and apprehension in social settings, often resulting in avoidance behaviors that can further diminish emotional self-efficacy (Kashdan et al., 2011). Individuals with heightened social interaction anxiety experience difficulties in establishing and maintaining meaningful social connections, exacerbating feelings of loneliness and further lowering their confidence in their emotional regulation (Robert Eres et al., 2021). This condition is particularly concerning in college students, as higher education environments frequently demand active participation in group discussions, social networking, and professional development activities (Xiao & Huang, 2022). The inability to engage effectively in these social settings may contribute to a vicious cycle wherein anxiety-induced withdrawal leads to increased loneliness, further reducing self-efficacy in emotional regulation (Maes et al., 2019).

Research suggests that social interaction anxiety plays a mediating role between loneliness and emotional self-efficacy, reinforcing the psychological distress associated with both constructs (Ghiggia et al., 2024). The presence of social interaction anxiety can disrupt emotional regulation by increasing self-doubt, leading individuals to perceive themselves as incapable of handling social and emotional challenges (Garke et al., 2025). Furthermore, heightened social anxiety has been linked to cognitive distortions and negative self-appraisals, which further erode self-efficacy beliefs and intensify emotional dysregulation (Özdemir & Kuru, 2023).

## 5. Current Study Aims and Hypotheses

To deepen the understanding of the connection between loneliness and regulatory emotional self-efficacy, recent work has aimed to disentangle the complex interactions among loneliness, life satisfaction, social interaction anxiety, and emotional self-efficacy (Dadfarnia et al., 2023; Jiang et al., 2020; Tu & Zhang, 2015). However, investigations into the mechanisms by which loneliness impacts emotional self-efficacy among university students, especially through the sequential mediating roles of life satisfaction and social interaction anxiety, are still limited. Consequently, this study aims to contribute to the existing body of research by exploring the pathways linking loneliness to regulatory emotional self-efficacy, emphasizing the chain mediation effects of life satisfaction and social interaction anxiety.

This study proposes four hypotheses:

**H1.**  *Loneliness is negatively associated with regulatory emotional self-efficacy, indicating that higher levels of perceived loneliness are likely to result in reduced confidence in emotional regulation abilities among university students.*

**H2.**  *Satisfaction with life mediates the relationship between loneliness and regulatory emotional self-efficacy, suggesting that loneliness negatively impacts satisfaction with life, which in turn hinders regulatory emotional self-efficacy.*

**H3.**  *Social interaction anxiety mediates the relationship between loneliness and regulatory emotional self-efficacy, suggesting that loneliness increases social interaction anxiety, which in turn reduces regulatory emotional self-efficacy.*

**H4.**  *Loneliness influences regulatory emotional self-efficacy through the chain-mediating effects of satisfaction with life and social interaction anxiety.*

**Figure 1:**
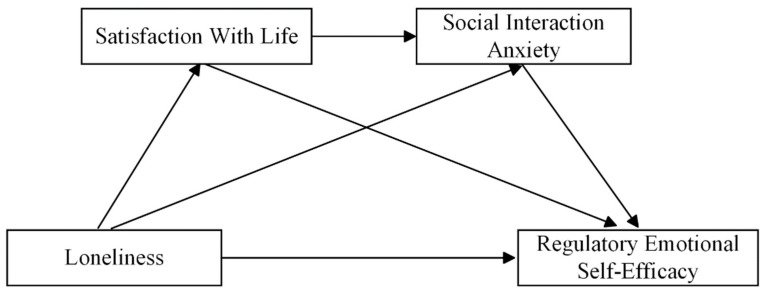
Chain mediation model.

By integrating SDT and SCT with Affect-as-Information Theory and Emotional Reasoning Theory, this theoretical model proposes that loneliness undermines regulatory emotional self-efficacy through the sequential mediating effects of satisfaction with life and social interaction anxiety. This process highlights the interaction between cognitive and emotional factors in influencing individuals’ ability to regulate emotions. Accordingly, this study introduces a chain mediation (see Figure 1) model to explore these connections and provide empirical support and theoretical validation for the idea that reducing loneliness can markedly improve regulatory emotional self-efficacy through the sequential influence of satisfaction with life and social interaction anxiety.

## 6. Research Subjects and Methods

### 6.1. Study Design

This study employed a cross-sectional design to examine the relationship between loneliness and regulatory emotional self-efficacy, with satisfaction with life and social interaction anxiety as mediators. The research was conducted at a university in Nanchang, Jiangxi Province, China. Ethical approval was obtained from the Institutional Review Board of Jiangxi University of Finance and Economics, ensuring compliance with relevant research guidelines.

### 6.2. Participants and Setting

#### 6.2.1. Sampling Strategy

A hybrid sampling strategy was utilized in this study, combining random sampling and cluster sampling techniques to recruit participants from a university located in Nanchang, Jiangxi Province.

#### 6.2.2. Sample Size and Validity

A total of 580 questionnaires were distributed among the student population, yielding 571 responses. After a thorough screening process to eliminate 24 incomplete questionnaires, 547 were confirmed as valid, achieving a validity rate of 95.797%. The valid sample included 246 male participants (44.973%) and 301 female participants (55.027%). Participants were categorized based on their academic year: 126 (23.035%) were in their first year, 197 (36.015%) in their second year, 113 (20.658%) in their third year, and 111 (20.293%) in their fourth year.

#### 6.2.3. Ethical Considerations

This study was approved by the ethics committee of Jiangxi University of Finance and Economics. All methods were performed in accordance with relevant guidelines and regulations. Informed consent was obtained from all participants prior to their inclusion in the study.

#### 6.2.4. Demographic Overview and Research Context

Table 1 provides a detailed demographic breakdown of the sample, offering a clear depiction of its composition. This demographic profile highlights the diversity of the participants and lays the foundation for an in-depth exploration of the relationships between loneliness, life satisfaction, social interaction anxiety, and emotional self-efficacy regulation within this group.

### 6.3. Variables and Measures

#### 6.3.1. Loneliness

The “ULS-6 Loneliness Scale”, revised by Zhou Liang and colleagues from Hays and DiMatteo’s original instrument and specifically tailored for Chinese populations, was employed to assess loneliness (Zhou et al., 2012). This single-dimension scale consists of six items and utilizes a four-point Likert scale, with higher scores approaching four indicating greater levels of loneliness. The scale exhibited strong internal consistency and reliability in this study, as indicated by a Cronbach’s alpha of 0.883. Confirmatory factor analysis (CFA) supported its structural validity, demonstrating a satisfactory model fit.

#### 6.3.2. Satisfaction with Life

For assessing satisfaction with life, this study utilized the “Satisfaction with Life Scale”, translated and revised into Chinese by Xiong and Xu based on the original scale developed by Diener et al. (Xiong & Xu, 2009). This single-dimension scale consists of five items and employs a seven-point Likert scale, with higher scores approaching seven indicating greater satisfaction with life. The scale’s internal consistency and reliability were validated in this study, as indicated by a Cronbach’s alpha of 0.846. Furthermore, its structural validity was confirmed through CFA, indicating an acceptable model fit.

#### 6.3.3. Social Interaction Anxiety

The “Social Interaction Anxiety Scale”, revised by Ye Dongmei from the original instrument developed by Mattick and Clarke, was employed to assess social interaction anxiety among Chinese college students (Ye et al., 1993). This unidimensional scale consists of 19 items and uses a five-point Likert response format. Higher scores, approaching 5, indicate greater levels of social interaction anxiety. The scale demonstrated high internal consistency and reliability, as indicated by a Cronbach’s alpha of 0.925, and robust structural validity.

#### 6.3.4. Regulatory Emotional Self-Efficacy

The “Regulatory Emotional Self-Efficacy Scale”, revised by Wen Shufeng based on the original instrument developed by Caprara and colleagues, was employed to assess emotional self-efficacy among Chinese university students (Wen et al., 2009). This scale consists of 12 items divided into three dimensions: self-efficacy in regulating positive emotions, self-efficacy in regulating depressed/distressed emotions, and self-efficacy in regulating angry/aggressive emotions. The scale uses a five-point Likert format, with higher scores (near five) reflecting greater emotional self-efficacy. It exhibited solid internal consistency and reliability, as evidenced by a Cronbach’s alpha of 0.873, and strong structural validity.

### 6.4. Research Methods

#### 6.4.1. Statistical Software and Analytical Techniques

The data analysis in this study was performed using SPSS 25.0 and Amos 26.0, encompassing assessments of reliability, validity, and common method bias, correlation analysis, regression analysis, and structural equation modeling.

#### 6.4.2. Assessment of Normality

Given the large sample size, the Kolmogorov–Smirnov (KS) test was employed to assess normality (Wood, 1978). The results showed no significant deviations from normality, as indicated by non-significant *p*-values; however, considering the KS test’s sensitivity to large samples, normality was further evaluated using skewness and kurtosis values, which fell within an acceptable range (Lee et al., 2019). These findings support the appropriateness of parametric statistical analyses.

#### 6.4.3. Mediation Analysis

Mediation effects were examined using the Bootstrap method with 5000 resamples, which did not assume normality, and their significance was determined by ensuring that the 95% confidence interval of the Bootstrap estimates did not include zero.

## 7. Results and Analysis

### 7.1. Common Method Bias Test

To ensure the validity of the results, this study tackled the potential issue of common method bias (CMB), a challenge in self-reported survey research that can skew the relationships between variables due to the use of a single data collection method (Kamakura, 2010). Proactive measures were taken during data collection, such as guaranteeing participant anonymity and including items designed to mitigate response biases. To further evaluate the presence of CMB, Harman’s single-factor test was employed as a post hoc diagnostic tool (Aguirre-Urreta & Hu, 2019). An exploratory factor analysis (EFA) of all items, performed without factor rotation, identified seven factors with eigenvalues exceeding 1, indicating a multidimensional structure. The largest factor explained 28.216% of the total variance, which is well below the commonly referenced 50% threshold for substantial CMB risk (Fuller et al., 2016).

These results suggest that common method bias is unlikely to have had a substantial impact on the data, thereby supporting the reliability and robustness of the study’s findings. This comprehensive approach underscores the methodological care taken to ensure the validity of the conclusions and the authenticity of the observed relationships among the variables.

### 7.2. Descriptive Statistics and Correlation Analysis

#### 7.2.1. Descriptive Statistics Overview

Descriptive statistics and correlation analyses were performed using SPSS 25.0. The results, which include means, standard deviations, and Pearson correlation coefficients, are presented in Table 2. This table provides an overview of the descriptive data and the interrelationships between the variables examined.

#### 7.2.2. Correlation Analysis

The findings reveal significant relationships among the key variables. Specifically, loneliness was positively correlated with social interaction anxiety (*r* = 0.486, *p* < 0.01), indicating that individuals experiencing higher levels of loneliness are more likely to report greater social interaction anxiety. In contrast, regulatory emotional self-efficacy was negatively associated with both loneliness (*r* = −0.363, *p* < 0.01) and social interaction anxiety (*r* = −0.410, *p* < 0.01), suggesting that individuals with stronger regulatory emotional self-efficacy tend to experience less loneliness and lower levels of social interaction anxiety. Furthermore, satisfaction with life was negatively correlated with loneliness (*r* = −0.350, *p* < 0.01) and positively associated with regulatory emotional self-efficacy (*r* = 0.504, *p* < 0.01), indicating that higher life satisfaction aligns with greater emotional regulation capabilities and reduced loneliness.

#### 7.2.3. Demographic Variables and Psychological Factors

No significant relationships were found between the demographic variables (e.g., gender, grade, only child status) and the psychological variables under study, except for a small but significant positive correlation between gender and social interaction anxiety (*r* = 0.093, *p* < 0.05). Overall, these results underscore the interplay between loneliness, satisfaction with life, social interaction anxiety, and emotional self-efficacy regulation, highlighting potential areas for targeted interventions to enhance emotional well-being.

### 7.3. Chain Mediation Effect Testing

A direct effects model connecting loneliness to regulatory emotional self-efficacy was initially proposed. The fit indices for the model were X^2^/df = 3.219, RMSEA = 0.054, CFI = 0.981, GFI = 0.984, and TLI = 0.973, demonstrating a good fit with the data and providing support for the first hypothesis.

Next, a chain mediation model incorporating satisfaction with life and social interaction anxiety as mediators was constructed. The model’s fit indices were X^2^/df = 3.259, RMSEA = 0.059, CFI = 0.976, GFI = 0.985, TLI = 0.960, and RMR = 0.030, indicating a strong alignment between the data and the model. Indirect effect models 1, 2, and 3 were developed, and the significance of the mediation effects was evaluated using bias-corrected percentile Bootstrap analysis, as summarized in Table 3. The sequences “Loneliness → Satisfaction With Life → Regulatory Emotional Self-Efficacy”, “Loneliness → Social Interaction Anxiety → Regulatory Emotional Self-Efficacy”, and “Loneliness → Satisfaction With Life → Social Interaction Anxiety → Regulatory Emotional Self-Efficacy” demonstrated significant indirect effects.

The standardized effect size for indirect effect 1 was −0.144, with a CI of [−0.189, −0.099]; for indirect effect 2, it was −0.120, with a CI of [−0.166, −0.078]; and for indirect effect 3, it was −0.009, with a CI of [−0.021, −0.002]. Since none of the confidence intervals included 0, the indirect effects were significant, thus supporting the second, third, and fourth hypotheses. Additionally, the direct effect of loneliness on regulatory emotional self-efficacy was significant (−0.097, CI of [−0.179, −0.014]), alongside a notable total indirect effect (−0.273, CI of [−0.334, −0.214]).

These results highlight the essential mediating roles of satisfaction with life and social interaction anxiety in linking loneliness to regulatory emotional self-efficacy, demonstrating a chain mediation effect. The results emphasize the importance of addressing loneliness and enhancing satisfaction with life and social interaction dynamics to improve emotional self-regulation.

## 8. Discussion

Drawing from prior research, this study proposed a model where loneliness acts as the predictor variable, satisfaction with life and social interaction anxiety serve as mediators, and regulatory emotional self-efficacy is the outcome variable, demonstrating a chain mediation effect. The key findings reveal that loneliness directly reduces regulatory emotional self-efficacy and that satisfaction with life mediates the link between loneliness and regulatory emotional self-efficacy. Similarly, social interaction anxiety also serves as a mediator in this relationship. Additionally, the combined mediating roles of satisfaction with life and social interaction anxiety reveal a sequential mediation effect linking loneliness to regulatory emotional self-efficacy. These findings offer a basis for further exploration and discussion. These findings align with previous research (Jin et al., 2020; Tan et al., 2022), providing a theoretical foundation for future interventions aimed at improving emotional self-regulation through targeted psychological and social support.

### 8.1. Direct Impact of Loneliness on Regulatory Emotional Self-Efficacy

The results reveal a significant negative relationship between loneliness and regulatory emotional self-efficacy. Additionally, the direct effect of loneliness on regulatory emotional self-efficacy is substantial, offering robust support for the first hypothesis. This conclusion aligns with previous studies by Jin et al. (2020) and Tan et al. (2022), both of which highlighted the adverse effects of loneliness on individuals’ confidence in managing their emotions.

In the context of increasing social isolation and the evolving challenges faced by university students, understanding the implications of loneliness has become increasingly important. University life often places students in situations that demand high levels of emotional resilience and self-regulation, making perceived loneliness a critical factor affecting their emotional well-being (Wang et al., 2024). As students navigate the complexities of academic and social interactions, the ability to effectively regulate emotions is indispensable for maintaining mental health and achieving personal and professional growth (Nadeem et al., 2023). To mitigate these negative effects, universities could implement peer mentoring programs and structured social activities that facilitate meaningful interpersonal connections, helping students build confidence in their emotional regulation skills.

### 8.2. Mediating Role of Satisfaction with Life

The standardized effect size for the indirect effect along Path 1, “Loneliness → Satisfaction With Life → Regulatory Emotional Self-Efficacy”, indicates that satisfaction with life significantly mediates the impact of loneliness on regulatory emotional self-efficacy. This finding suggests that satisfaction with life serves as a critical intermediary, whereby loneliness diminishes life satisfaction, which subsequently undermines regulatory emotional self-efficacy, confirming the validity of the second hypothesis.

These findings deepen our understanding of the emotional dynamics experienced by university students, highlighting the essential role of satisfaction with life in mitigating the adverse effects of loneliness on emotional regulation. University life often brings significant stressors, including social isolation, academic pressure, and transitions to independence, which can amplify loneliness and reduce overall life satisfaction (Aslan & Polat, 2024). This diminished life satisfaction, in turn, hinders students’ confidence in their ability to manage emotions effectively, further affecting their capacity to navigate the complexities of university life. Ultimately, this finding calls for a more integrative approach in higher education, combining support for emotional well-being with academic guidance. By prioritizing efforts to reduce loneliness and enhance satisfaction with life, universities can empower students to develop stronger regulatory emotional self-efficacy, better equipping them for both academic success and personal growth. This integrated perspective supports the development of a well-rounded and resilient student body capable of thriving in the face of the multifaceted challenges of higher education and beyond.

### 8.3. Mediating Role of Social Interaction Anxiety

The standardized effect size for the indirect effect along Path 2, “Loneliness → Social Interaction Anxiety → Regulatory Emotional Self-Efficacy”, shows that loneliness significantly influences regulatory emotional self-efficacy through social interaction anxiety. This finding confirms that social interaction anxiety mediates the link between loneliness and regulatory emotional self-efficacy, supporting the validity of the third hypothesis.

In the university context, loneliness often amplifies feelings of discomfort and apprehension during social interactions, which are key components of social interaction anxiety. This heightened anxiety can undermine students’ confidence in their ability to regulate emotions effectively, particularly in social or academic settings. The presence of social interaction anxiety creates a cycle where students may avoid or withdraw from interactions that could otherwise enhance their emotional regulation skills, further compounding the impact of loneliness on their emotional well-being.

Given these findings, universities should consider implementing targeted interventions, such as social skills training workshops or cognitive behavioral therapy (CBT) programs, to help students overcome social interaction anxiety. Research suggests that CBT interventions can significantly reduce social anxiety symptoms and improve self-efficacy in managing emotions (Kivity et al., 2021). These structured approaches may be particularly effective in fostering a more socially supportive and emotionally resilient student body.

### 8.4. The Chain-Mediating Role of Satisfaction with Life and Social Interaction Anxiety

The standardized effect size for the indirect effect along Path 3, “Loneliness → Satisfaction With Life → Social Interaction Anxiety → Regulatory Emotional Self-Efficacy”, reveals that loneliness significantly impacts regulatory emotional self-efficacy through the sequential mediation of satisfaction with life and social interaction anxiety. This finding validates the proposed fourth hypothesis, underscoring the intricate role of satisfaction with life and social interaction anxiety as chain mediators in this relationship. It highlights the broader impact of loneliness, which extends beyond a simple emotional state to influence broader psychological processes, including self-regulation and emotional efficacy.

Understanding this multi-layered mediation pathway is essential for developing interventions aimed at mitigating loneliness and its broader psychological consequences. Specifically, addressing both life satisfaction and social interaction anxiety could provide a dual-targeted approach to enhance individuals’ regulatory emotional self-efficacy. This perspective reinforces the necessity for strategies that integrate emotional and social support, emphasizing the importance of fostering environments that cultivate life satisfaction and reduce social interaction anxiety among undergraduate students. This expanded understanding of the mediating effects of satisfaction with life and social interaction anxiety calls for a more nuanced approach to tackling loneliness, advocating for comprehensive psychological and social interventions. These initiatives should focus on establishing supportive community frameworks that tackle both the emotional and social aspects of loneliness, thereby fostering the comprehensive growth of students’ emotional self-efficacy and overall well-being.

## 9. Recommendations and Strategies

### 9.1. Reducing Loneliness to Foster Emotional Regulation in University Settings

Loneliness is a prevalent issue among university students, often stemming from the transition to a new environment and the challenges of building social connections. Research highlights that structured social programs, such as mentorship initiatives and orientation events, significantly reduce student loneliness by fostering peer support and integration (Amanvermez et al., 2023). These interventions not only help students establish meaningful relationships but also contribute to enhancing emotional regulation, as social support has been linked to improved emotional well-being and stress management.

To address loneliness and strengthen students’ ability to regulate their emotions, universities should prioritize structured peer interaction programs, including student clubs and community engagement initiatives (Ellard et al., 2023). Additionally, accessible psychological support systems, including counseling centers offering both individual and group therapy, can help students manage feelings of isolation while also developing essential emotional regulation strategies. Digital platforms can complement in-person interactions by providing online communities where students can connect and share experiences, fostering both social bonding and emotional resilience.

Encouraging participation in campus-wide social initiatives and providing spaces for informal gatherings can further mitigate loneliness, enabling students to develop the stronger emotional regulation skills necessary to cope with academic and personal challenges. By addressing loneliness through targeted interventions, universities can empower students to navigate university life with improved emotional well-being and self-efficacy.

### 9.2. Enhancing Life Satisfaction to Build Resilience Among University Students

Life satisfaction is a key factor influencing students’ well-being and academic achievement. Research indicates that interventions such as mindfulness-based practices, recreational activities, and goal-setting workshops contribute to greater life satisfaction among students (Bamber & Schneider, 2016). Universities can enhance students’ satisfaction with life by promoting activities aligned with their interests and personal goals. Offering workshops on personal development and stress management can provide students with a sense of purpose and direction. Moreover, integrating mindfulness practices, such as meditation and yoga sessions, into campus life has been shown to cultivate gratitude and enhance emotional regulation (Tolbaños-Roche & Menon, 2021). By fostering life satisfaction, universities empower students to approach challenges with resilience and optimism, further enhancing their academic performance.

### 9.3. Addressing Social Interaction Anxiety to Improve Student Confidence

Social interaction anxiety is a significant barrier to forming meaningful connections and fully engaging in campus life. Studies suggest that cognitive behavioral therapy (CBT) and social skills training programs effectively reduce social anxiety and improve self-efficacy in communication (O’Shannessy et al., 2023). Universities should implement targeted support programs, such as public speaking workshops and interpersonal communication training, to help students navigate social interactions confidently. Counseling centers can offer CBT-based group therapy tailored to social anxiety. Additionally, peer mentoring programs, where upper-year students support new students, can create a sense of belonging and reduce the intimidation of social engagement (Guo et al., 2025). Low-risk social activities, such as meet-and-greet events or interest-based clubs, allow students to gradually build confidence in social settings (Chapin et al., 2019). Addressing social interaction anxiety not only strengthens interpersonal skills but also contributes to overall well-being and academic success (Habibi Asgarabad et al., 2023).

### 9.4. Strengthening Regulatory Emotional Self-Efficacy for Academic and Personal Growth

RESE is crucial for students navigating the emotional challenges of university life. Universities should incorporate RESE-focused training into academic and extracurricular activities. Workshops on stress management, emotional intelligence, and coping strategies can equip students with practical tools for managing their emotions. Integrating RESE skills into academic curricula, such as through reflective journaling exercises or collaborative projects, allows students to practice and develop these competencies (Chen et al., 2024). Additionally, universities can provide students with digital tools, such as mobile apps for tracking their mood and managing stress, enabling them to monitor and regulate their emotional states in real time (Eisenstadt et al., 2021). By fostering RESE, universities prepare students to handle academic pressures, interpersonal conflicts, and future career challenges with greater confidence and resilience.

### 9.5. Creating Integrated Campus-Based Interventions for Holistic Development

To address the interconnected issues of loneliness, life satisfaction, social interaction anxiety, and emotional regulation, universities should adopt a comprehensive and integrated approach. This involves combining social, emotional, and academic support systems to create a cohesive framework for student well-being. Collaboration among student affairs offices, counseling centers, academic advisors, and student organizations is essential for designing interventions that address the diverse needs of the student population (Leary et al., 2022). For example, a “Wellness Week” could combine stress management workshops, social mixers, and life satisfaction seminars to tackle multiple aspects of student well-being simultaneously (Bleck et al., 2023). Universities should also foster a campus culture that values mental health by providing easily accessible resources, reducing the stigma associated with seeking help, and training faculty and staff to recognize and support students in distress (Withers et al., 2022). Such integrated strategies ensure a supportive environment where students can thrive academically, socially, and emotionally, preparing them for success in both university and post-graduation life.

## 10. Conclusions

This study examined the relationship between loneliness and regulatory emotional self-efficacy, with satisfaction with life and social interaction anxiety serving as mediating variables. The findings indicate that loneliness negatively impacts regulatory emotional self-efficacy both directly and indirectly through the mediating effects of satisfaction with life and social interaction anxiety. Notably, satisfaction with life and social interaction anxiety also function as sequential mediators, highlighting a complex interplay between cognitive and emotional factors in shaping individuals’ emotional self-regulation.

These results underscore the importance of addressing loneliness among university students to enhance their emotional self-efficacy. Interventions aimed at improving life satisfaction and reducing social interaction anxiety may serve as effective strategies for mitigating the negative effects of loneliness on emotional regulation. Future research should explore additional psychosocial variables and employ longitudinal designs to further elucidate the long-term implications of these relationships. By fostering supportive social environments and promoting psychological well-being, institutions can contribute to the overall resilience and academic success of students.

## 11. Limitations and Future Studies

First, the reliance on self-reported questionnaires presents inherent limitations, as individual benchmarks and subjective interpretations may introduce biases. Participants’ self-assessments of loneliness, life satisfaction, social interaction anxiety, and regulatory emotional self-efficacy are influenced by personal perceptions, potentially compromising the accuracy and reliability of the data. This methodological constraint underscores the need for future studies to incorporate multi-method approaches, such as peer evaluations, behavioral observations, or physiological measures, to triangulate findings and minimize subjective biases.

Second, the study concentrated solely on university students from one institution in Nanchang, Jiangxi Province, potentially restricting the generalizability of the findings. Although the demographic variety within the sample offers meaningful insights, the geographic and institutional specificity limits the broader applicability of the results. Future research should expand sampling efforts to include universities across various regions and contexts, ensuring a more representative depiction of the relationships among loneliness, life satisfaction, social interaction anxiety, and regulatory emotional self-efficacy.

Third, the scope of the variables examined, while comprehensive in certain respects, may have overlooked critical dimensions. For instance, while the study emphasized positive mediating factors such as life satisfaction, negative outcomes like maladaptive emotional regulation strategies or broader mental health concerns were not explored. Addressing these limitations in future research by investigating both adaptive and maladaptive pathways would offer a more balanced and holistic perspective.

These identified limitations suggest significant opportunities for methodological enhancement and theoretical expansion. Improving measurement methods, expanding participant selection to encompass a wider range of regional and institutional settings, and including a broader array of variables will help develop a more comprehensive understanding of the relationships between loneliness, life satisfaction, social interaction anxiety, and regulatory emotional self-efficacy. Such efforts will deepen insights into these dynamics, providing a stronger foundation for interventions that address the multifaceted challenges faced by university students.

## Figures and Tables

**Table 1:** Demographic characteristics.

Variable	Category	Number of Participants	Proportion (%)
Gender	Male	246	44.973
	Female	301	55.027
Grade	Freshman	126	23.035
	Sophomore	197	36.015
	Junior	113	20.658
	Senior	111	20.293
Only Child Status	Yes	244	44.607
	No	303	55.393
Place of Origin	Rural	239	43.693
	Urban	308	56.307

**Table 2:** Descriptive statistics and correlation analysis.

	Mean	SD	1	2	3	4	5	6	7	8
1 Gender	1.550	0.498	1							
2 Grade	2.382	1.051	−0.067	1						
3 Only Child	1.554	0.498	0.076	0.011	1					
4 Place of Origin	1.437	0.496	0.033	0.045	0.449 **	1				
5 Loneliness	2.100	0.668	0.074	0.031	0.110 **	0.062	1			
6 Satisfaction With Life	4.354	1.259	0.006	0.010	−0.116 **	−0.151 **	−0.350 **	1		
7 Social Interaction Anxiety	2.750	0.797	0.093 *	0.010	0.073	0.073	0.486 **	−0.255 **	1	
8 Regulatory Emotional Self-Efficacy	3.511	0.680	−0.042	0.045	−0.077	−0.04	−0.363 **	0.504 **	−0.410 **	1

N = 547; * *p* < 0.05, ** *p* < 0.01.

**Table 3:** Bootstrap analysis to assess the significance of mediation effects (N = 547).

Path	Standardized Effect Size	95% CI
Direct Effect: Loneliness → Regulatory Emotional Self-Efficacy	−0.097	[−0.179, −0.014]
Total Indirect Effect	−0.273	[−0.334, −0.214]
Indirect Effect 1: Loneliness → Satisfaction With Life → Regulatory Emotional Self-Efficacy	−0.144	[−0.189, −0.099]
Indirect Effect 2: Loneliness → Social Interaction Anxiety → Regulatory Emotional Self-Efficacy	−0.120	[−0.166, −0.078]
Indirect Effect 3: Loneliness → Satisfaction With Life → Social Interaction Anxiety → Regulatory Emotional Self-Efficacy	−0.009	[−0.021, −0.002]

## Data Availability

The data presented in this study are available on request from the corresponding author due to the rules and regulations of the Jiangxi University of Finance and Economics Ethics Committee.

## References

[B1-behavsci-15-00392] Acharya L., Jin L., Collins W. (2018). College life is stressful today–Emerging stressors and depressive symptoms in college students. Journal of American College Health.

[B2-behavsci-15-00392] Aguirre-Urreta M. I., Hu J. (2019). Detecting common method bias: Performance of the harman’s single-factor test. SIGMIS Database.

[B3-behavsci-15-00392] Amanvermez Y., Rahmadiana M., Karyotaki E., de Wit L., Ebert D. D., Kessler R. C., Cuijpers P. (2023). Stress management interventions for college students: A systematic review and meta-analysis. Clinical Psychology: Science and Practice.

[B4-behavsci-15-00392] Aslan I., Polat H. (2024). Investigating social media addiction and impact of social media addiction, loneliness, depression, life satisfaction and problem-solving skills on academic self-efficacy and academic success among university students. Frontiers in Public Health.

[B5-behavsci-15-00392] Bacon T., Doughty C., Summers A., Wiffen B., Stanley Z., McAlpine S. (2018). The emotional resources group: Provisional outcome data for a pilot six-session emotion regulation programme for secondary care. British Journal of Clinical Psychology.

[B6-behavsci-15-00392] Bamber M. D., Schneider J. K. (2016). Mindfulness-based meditation to decrease stress and anxiety in college students: A narrative synthesis of the research. Educational Research Review.

[B7-behavsci-15-00392] Beiter R., Nash R., McCrady M., Rhoades D., Linscomb M., Clarahan M., Sammut S. (2015). The prevalence and correlates of depression, anxiety, and stress in a sample of college students. Journal of Affective Disorders.

[B8-behavsci-15-00392] Bleck J., DeBate R., Garcia J., Gatto A. (2023). A pilot evaluation of a university health and wellness coaching program for college students. Health Education & Behavior.

[B9-behavsci-15-00392] Cacioppo J. T., Hawkley L. C., Crawford L. E., Ernst J. M., Burleson M. H., Kowalewski R. B., Malarkey W. B., Van Cauter E., Berntson G. G. (2002). Loneliness and health: Potential mechanisms. Psychosomatic Medicine.

[B10-behavsci-15-00392] Caprara G. V., Di Giunta L., Eisenberg N., Gerbino M., Pastorelli C., Tramontano C. (2008). Assessing regulatory emotional self-efficacy in three countries. Psychological Assessment.

[B11-behavsci-15-00392] Caprara M., Di Giunta L., Bermúdez J., Caprara G. V. (2020). How self-efficacy beliefs in dealing with negative emotions are associated to negative affect and to life satisfaction across gender and age. PLoS ONE.

[B12-behavsci-15-00392] Chapin L. A., Deans C. L., Fabris M. A. (2019). “After film club, I actually got better at everything”: School engagement and the impact of an after-school film club. Children and Youth Services Review.

[B13-behavsci-15-00392] Chen P., Yang D., Lavonen J., Metwally A. H. S., Tang X. (2024). How do students of different self-efficacy regulate learning in collaborative design activities? An epistemic network analysis approach. Frontiers in Psychology.

[B14-behavsci-15-00392] Clore G. L., Gasper K., Garvin E. (2012). Affect as information. Handbook of affect and social cognition.

[B15-behavsci-15-00392] Cui L., Criss M. M., Ratliff E., Wu Z., Houltberg B. J., Silk J. S., Morris A. S. (2020). Longitudinal links between maternal and peer emotion socialization and adolescent girls’ socioemotional adjustment. Developmental Psychology.

[B16-behavsci-15-00392] Dadfarnia S., Taherifar Z., Farahani H. (2023). Emotion regulation strategies as a mediator of the relationship of beliefs about emotion and emotion regulation self-efficacy, and social anxiety. Practice in Clinical Psychology.

[B17-behavsci-15-00392] Dagan Y., Yager J. (2019). Addressing loneliness in **complex PTSD**. The Journal of Nervous and Mental Disease.

[B18-behavsci-15-00392] Diener E., Emmons R. A., Larsen R. J., Griffin S. (1985). The satisfaction with life scale. Journal of Personality Assessment.

[B19-behavsci-15-00392] Eisenstadt M., Liverpool S., Infanti E., Ciuvat R. M., Carlsson C. (2021). Mobile apps that promote emotion regulation, positive mental health, and well-being in the general population: Systematic review and meta-analysis. JMIR Mental Health.

[B20-behavsci-15-00392] Elegbede P. T., Ogunleye A. J. (2018). Emotional control, self-efficacy and social support as predictors of intimate relationship satisfaction among dating partners. IFE PsychologIA: An International Journal.

[B21-behavsci-15-00392] Ellard O. B., Dennison C., Tuomainen H. (2023). Review: Interventions addressing loneliness amongst university students: A systematic review. Child and Adolescent Mental Health.

[B22-behavsci-15-00392] Fromm-Reichmann F. (1990). Loneliness. Contemporary Psychoanalysis.

[B23-behavsci-15-00392] Fuller C. M., Simmering M. J., Atinc G., Atinc Y., Babin B. J. (2016). Common methods variance detection in business research. Journal of Business Research.

[B24-behavsci-15-00392] Gangemi A., Mancini F., Johnson-Laird P. N. (2013). Emotion, reasoning, and psychopathology. Emotion and Reasoning.

[B25-behavsci-15-00392] Garke M. Å., Hentati Isacsson N., Kolbeinsson Ö., Hesser H., Månsson K. N. (2025). Improvements in emotion regulation during cognitive behavior therapy predict subsequent social anxiety reductions. Cognitive Behaviour Therapy.

[B26-behavsci-15-00392] Ghiggia A., Castelli L., Adenzato M., Di Tella M. (2024). Emotional competencies and psychological distress: Is loneliness a mediating factor?. Scandinavian Journal of Psychology.

[B27-behavsci-15-00392] Gómez A. S. (2020). Psychosocial factors and clinical predictors of suicide risk in college students. Mediterranean Journal of Clinical Psychology.

[B28-behavsci-15-00392] Guo J., Talib M. A., Guo B., Ren J. (2025). Smartphone addiction as a moderator of undergraduates’ sense of coherence, social support, and satisfaction with life. Social Behavior and Personality.

[B29-behavsci-15-00392] Habibi Asgarabad M., Steinsbekk S., Wichstrøm L. (2023). Social skills and symptoms of anxiety disorders from preschool to adolescence: A prospective cohort study. Journal of Child Psychology and Psychiatry.

[B30-behavsci-15-00392] Hayes S., Carlyle M., Haslam S. A., Haslam C., Dingle G. (2022). Exploring links between social identity, emotion regulation, and loneliness in those with and without a history of mental illness. British Journal of Clinical Psychology.

[B31-behavsci-15-00392] Hladek M. D., Nersesian P. V., Cudjoe T. K., Gill J. M., Szanton S. L. (2019). Higher coping self-efficacy associated with low self-perceived loneliness in older adults with chronic disease. Innovation in Aging.

[B32-behavsci-15-00392] Hyland P., Shevlin M., Cloitre M., Karatzias T., Vallières F., McGinty G., Fox R., Power J. M. (2019). Quality not quantity: Loneliness subtypes, psychological trauma, and mental health in the US adult population. Social Psychiatry and Psychiatric Epidemiology.

[B33-behavsci-15-00392] Jiang L., Liao M., Ying R. (2020). The relationship between loneliness, self-efficacy, and satisfaction with life in left-behind middle school students in china: Taking binhai county of jiangsu province as an example. Best Evidence in Chinese Education.

[B34-behavsci-15-00392] Jin Y., Zhang M., Wang Y., An J. (2020). The relationship between trait mindfulness, loneliness, regulatory emotional self-efficacy, and subjective well-being. Personality and Individual Differences.

[B35-behavsci-15-00392] Kamakura W. A. (2010). Common methods bias. Wiley international encyclopedia of marketing.

[B36-behavsci-15-00392] Karyotaki E., Cuijpers P., Albor Y., Alonso J., Auerbach R. P., Bantjes J., Bruffaerts R., Ebert D. D., Hasking P., Kiekens G., Lee S., McLafferty M., Mak A., Mortier P., Sampson N. A., Stein D. J., Vilagut G., Kessler R. C. (2020). Sources of stress and their associations with mental disorders among college students: Results of the world health organization world mental health surveys international college student initiative. Frontiers in Psychology.

[B37-behavsci-15-00392] Kashdan T. B., Weeks J. W., Savostyanova A. A. (2011). Whether, how, and when social anxiety shapes positive experiences and events: A self-regulatory framework and treatment implications. Clinical Psychology Review.

[B38-behavsci-15-00392] Kivity Y., Cohen L., Weiss M., Elizur J., Huppert J. D. (2021). The role of expressive suppression and cognitive reappraisal in cognitive behavioral therapy for social anxiety disorder: A study of self-report, subjective, and electrocortical measures. Journal of Affective Disorders.

[B39-behavsci-15-00392] Kleef G. A. V. (2009). How emotions regulate social life: The emotions as social information (EASI) model. Current Directions in Psychological Science.

[B40-behavsci-15-00392] Leary M., Muller T. M., Kramer S., Sopper J., Gebauer R. D., Wade M. E. (2022). Defining collaboration through the lens of a Delphi study: Student affairs and academic affairs partnerships in residential learning communities. The Qualitative Report.

[B41-behavsci-15-00392] Lee S., Lee G., Jeon G. (2019). Statistical approaches based on deep learning regression for verification of normality of blood pressure estimates. Sensors.

[B42-behavsci-15-00392] Loeb C., Stempel C., Isaksson K. (2016). Social and emotional self-efficacy at work. Scandinavian Journal of Psychology.

[B43-behavsci-15-00392] Maes M., Nelemans S. A., Danneel S., Fernández-Castilla B., Van den Noortgate W., Goossens L., Vanhalst J. (2019). Loneliness and social anxiety across childhood and adolescence: Multilevel meta-analyses of cross-sectional and longitudinal associations. Developmental Psychology.

[B44-behavsci-15-00392] Malafanti A., Yotsidi V., Giannouli E., Paraskevadaki E., Malogiannis I. (2024). Concurrent associations between trauma and borderline personality organization in emerging adulthood. Mediterranean Journal of Clinical Psychology.

[B45-behavsci-15-00392] Mayiwar L., Hærem T., Løhre E. (2024). Self-distancing regulates the effect of incidental anger (vs. fear) on affective decision-making under uncertainty. Journal of Behavioral Decision Making.

[B46-behavsci-15-00392] Meng J., Wang X., Wei D., Qiu J. (2020). State loneliness is associated with emotional hypervigilance in daily life: A network analysis. Personality and Individual Differences.

[B47-behavsci-15-00392] Nadeem A., Umer F., Anwar M. J. (2023). Emotion regulation as predictor of academic performance in university students. Journal of Professional & Applied Psychology.

[B48-behavsci-15-00392] O’Shannessy D. M., Waters A. M., Donovan C. L. (2023). Feasibility of an intensive, disorder-specific, group-based cognitive behavioural therapy intervention for adolescents with social anxiety disorder. Child Psychiatry & Human Development.

[B49-behavsci-15-00392] Özdemir İ., Kuru E. (2023). Investigation of cognitive distortions in panic disorder, generalized anxiety disorder and social anxiety disorder. Journal of Clinical Medicine.

[B50-behavsci-15-00392] Pocnet C., Dupuis M., Congard A., Jopp D. (2017). Personality and its links to quality of life: Mediating effects of emotion regulation and self-efficacy beliefs. Motivation and Emotion.

[B51-behavsci-15-00392] Ramón-Arbués E., Gea-Caballero V., Granada-López J. M., Juárez-Vela R., Pellicer-García B., Antón-Solanas I. (2020). The prevalence of depression, anxiety and stress and their associated factors in college students. International Journal of Environmental Research and Public Health.

[B52-behavsci-15-00392] Robert Eres C. J., Lim M. H., Lanham S., Bates G. (2021). Loneliness and emotion regulation: Implications of having social anxiety disorder. Australian Journal of Psychology.

[B53-behavsci-15-00392] Ryan R. M., Deci E. L. (2000). Self-determination theory and the facilitation of intrinsic motivation, social development, and well-being. American Psychologist.

[B54-behavsci-15-00392] Schunk D. H., DiBenedetto M. K. (2020). Motivation and social cognitive theory. Contemporary Educational Psychology.

[B55-behavsci-15-00392] Tan A. J., Mancini V., Gross J. J., Goldenberg A., Badcock J. C., Lim M. H., Becerra R., Jackson B., Preece D. A. (2022). Loneliness versus distress: A comparison of emotion regulation profiles. Behaviour Change.

[B56-behavsci-15-00392] Tolbaños-Roche L., Menon P. (2021). Applying the S-ART framework to yoga: Exploring the self-regulatory action of yoga practice in two culturally diverse samples. Frontiers in Psychology.

[B57-behavsci-15-00392] Tsarpalis-Fragkoulidis A., van Eickels R. L., Zemp M. (2022). Please don’t compliment me! Fear of positive evaluation and emotion regulation—Implications for adolescents’ social anxiety. Journal of Clinical Medicine.

[B58-behavsci-15-00392] Tu Y., Zhang S. (2015). Loneliness and subjective well-being among chinese undergraduates: The mediating role of self-efficacy. Social Indicators Research.

[B59-behavsci-15-00392] Van Lange P., Kruglanski A., Higgins E., Schwarz N. (2012). Feelings-as-information theory. Handbook of Theories of Social Psychology.

[B60-behavsci-15-00392] Vansteenkiste M., Ryan R. M. (2013). On psychological growth and vulnerability: Basic psychological need satisfaction and need frustration as a unifying principle. Journal of Psychotherapy Integration.

[B61-behavsci-15-00392] Veronese G., Pepe A., Cavazzoni F., Obaid H., Perez J. (2019). Agency via life satisfaction as a protective factor from cumulative trauma and emotional distress among bedouin children in palestine. Frontiers in Psychology.

[B62-behavsci-15-00392] Wang Q., Yan G., Hu Y., Ding G., Lai Y. (2024). Stress and emotion in a locked campus: The moderating effects of resilience and loneliness. Frontiers in Psychology.

[B63-behavsci-15-00392] Wen S., Tang D., Yu G. (2009). The characteristics of regulatory emotional self-efficacy in Chinese graduate students. Psychological Science (China).

[B64-behavsci-15-00392] Withers M., Jahangir T., Kubasova K., Ran M.-S. (2022). Reducing stigma associated with mental health problems among university students in the Asia-Pacific: A video content analysis of student-driven proposals. International Journal of Social Psychiatry.

[B65-behavsci-15-00392] Wood C. L. (1978). A large-sample Kolmogorov-Smirnov test for normality of experimental error in a randomized block design. Biometrika.

[B66-behavsci-15-00392] Wu R., Jing L., Liu Y., Wang H., Yang J. (2022). Effects of physical activity on regulatory emotional self-efficacy, resilience, and emotional intelligence of nurses during the COVID-19 pandemic. Frontiers in Psychology.

[B67-behavsci-15-00392] Xiao Z., Huang J. (2022). The relation between college students’ social anxiety and mobile phone addiction: The mediating role of regulatory emotional self-efficacy and subjective well-being. Frontiers in Psychology.

[B68-behavsci-15-00392] Xiong C., Xu Y. (2009). Reliability and validity of the satisfaction with life scale for chinese demos. China Journal of Health Psychology.

[B69-behavsci-15-00392] Ye D., Qian M., Liu X., Chen X. (1993). Revision of social interaction anxiety scale and social phobia scale. Chinese Journal of Clinical Psychology.

[B70-behavsci-15-00392] Zafar M., Ansari K. (2020). Sleep disorders among undergraduate health students in Bristol, United Kingdom. Mediterranean Journal of Clinical Psychology.

[B71-behavsci-15-00392] Zhou L., Li Z., Hu M., Xiao S. (2012). Reliability and validity of ULS-8 loneliness scale in elderly samples in a rural community. Journal of Central South University. Medical Sciences.

